# Effects of applying a multivariate training program on physical fitness and tactical performance in a team sport taught during physical education classes

**DOI:** 10.3389/fspor.2023.1291342

**Published:** 2023-11-10

**Authors:** Avelino Silva, Ricardo Ferraz, Luís Branquinho, Tatiana Dias, José E. Teixeira, Daniel A. Marinho

**Affiliations:** ^1^ Sport Sciences Department, University of Beira Interior, Covilhã, Portugal; ^2^ Research Center in Sports Sciences, Health Sciences and Human Development, Covilhã, Portugal; ^3^ Agrarian School of Elvas, Polytechnic Institute of Portalegre, Elvas, Portugal; ^4^ Sport Department, Polytechnic Institute of Bragança, Bragança, Portugal; ^5^ Sport Department, Polytechnic Institute of Guarda, Guarda, Portugal; ^6^ CI-ISCE – ISCE Douro, Penafiel, Portugal

**Keywords:** multivariate program, tactical performance, physical fitness, school, basketball

## Abstract

**Introduction:**

A multivariate training program could be a pedagogical choice to improve physical and tactical performance in a team sport taught during physical education classes at different levels of education. Thus, the aim of this study was to verify the effects of applying a multivariate training program on physical fitness and tactical performance during the teaching of a basketball didactic unit in basic and secondary education.

**Methods:**

Seventy-five students from a Portuguese school, with an average age of 15.02 ± 1.31 years, included forty-two students from basic school and thirty-three students from secondary school. The FITescola® test battery was used to assess physical fitness (i.e., sit-ups, push-ups, horizontal impulse, shuttle test, 40 m sprint, agility 4 × 10 m). The Game Performance Assessment Instrument (GPAI) was used to assess students' tactical performance for each player's game performance during a 20-minute 3 vs. 3 match. The GPAI variables were decision making index (DMI), skill execution index (SEI), support actions index (SI), and adaptability index (AI). During a basketball didactic unit teaching, the students were randomly divided into two groups, a control group that will not carry out the training program and an experimental group that will carry out a strength training program, high intensity explosive exercises and activities based on small-sided games (SSG) for 6 weeks. The two groups were evaluated in two moments: before the application of the training program and after the application of the training program regarding changes in physical fitness and tactical performance. The independent samples t-test (samples from two groups) and paired sample Test (for the same group) were applied for pre and post-assessment comparisons.

**Results:**

All indexes present significant differences between basic and secondary students in the pre- and post-assessment tests with small effects (*t* = −6.54 to −4.82, Δ = −27.57 to −0.16, *p*<0.05–*p*< 0.001, *d* = 0.78–1.05).

**Discussion:**

The results allow to conclude that in a school environment, a well-structured multivariate training program can effectively improve students' tactical skills, increasing their physical conditioning levels.

## 1. Introduction

Today, physical education plays a key role in promoting active lifestyles through physical activity (1, 2). This discipline brings together many areas of knowledge, such as the development of sporting performance, the acquisition of specific technical skills, and the promotion a more active and healthier lifestyle (3). In fact, the benefits of regular practice of physical activity are many and varied, focusing on improving cardiovascular and respiratory function, reducing levels of anxiety and depression, increasing well-being and developing cognitive and social skills (4). In addition to the opportunity to express their motor skills, many students also emphasize the potential of physical education to satisfy their psychological needs through its differentiated practical nature (5). In fact, recent evidence supports the idea that physical fitness has a positive impact on mental health and quality of life (6, 7). Therefore, to ensure student engagement, it is essential that curriculum content is presented in an attractive way that is challenging and increases the likelihood of improving motor skills (8, 9).

It should be noted that in order to achieve the potential goals of physical education classes and to increase the associated benefits, the intensity of practice plays a preponderant role (10). It is therefore important to clarify the factors that determine whether students reach the desired level of intensity. The literature reports that at least 50% of the session should be planned as moderate to vigorous intensity physical activity (11). However, most of the time the intensity of the classes does not reach the recommended values (12). In this regard, other research suggests that lessons based on team games seem to induce higher intensities than sessions that include individual games and activities of an analytical nature (13, 14). However, high levels of motor competence have also been associated with the ability to perform tasks at higher intensities (15). The evidence seems to indicate that the use of collective games combined with the development of motor skills can help to achieve the proposed objectives. Furthermore, the physical fitness profile of students is related to their technical and tactical performance, which is a strong indicator of success in team games (16). Hence the importance of planning classes that recognize this relationship (17).

In this sense, multivariate training programs have been widely recognized as an effective strategy with a view for developing the skills necessary for good performance in the collective modalities addressed in physical education (10, 18), such as technical and tactical skills (19, 20). They prescribe functional loads that allow the students to overcome their typical muscle activity (21, 22). Typically, this type of program consists of multiple stations designed to improve strength, balance, endurance, coordination (23) and cardiovascular function (24). It has been recommended in combination with neuromuscular training, which includes regular activities for basic movement, specialized activities with exercises designed to improve motor deficits and physical conditioning exercises (directly affecting resistance, dynamic stability, plyometrics and agility) (25, 26). Furthermore, Fort-Vanmeerhaeghe et al. (27) highlight the need to implement an integrative neuromuscular training program to increase injury resistance and improve athletic and motor performance capabilities in young people. According to the authors, this is important because the consequences of a sedentary lifestyle during childhood and adolescence appear to have long-term effects on overall health, extending into adulthood, if unhealthy habits are not addressed and prevented during this critical period of life.

This type of program can also have some social and psychological benefits for young people, such as improving self-esteem and reducing depressive symptoms (24). Furthermore, its implementation has the potential to promote the development of both motor skills and creativity simultaneously (28), which can be particularly helpful in team sports. Essentially, this specific training program aims to help young people develop a strong foundation, improve their physical movements and mechanics, and build confidence in their physical abilities. It involves a well-rounded approach with a variety of exercises, gradual progression, and adequate rest for optimal recovery (25). This approach seems to provide the necessary tools to experiment, improve and master basic movements (i.e., locomotion, stability and manipulation skills) (21, 22, 27, 29). A previous study showed improvements in basic motor skills and physical fitness after an 8-week integrated neuromuscular training program, during the first part of the physical education class (i.e., 15 min) (30). Similar findings were documented in a recent study (31) that examined the effects of 10 weeks of integrated neuromuscular training in a school setting, while another investigation (32) examined the effects of combined integrated neuromuscular training with yoga and various stretching techniques.

In this sense, innovative and high-quality pedagogical interventions in physical education classes can be essential for the development of children's motor skills (33), which are necessary for learning collective modalities. The collective modalities have always had a prominent place in the school environment, mainly due to the receptivity of the students, because of their ease of application in the school context. However, the pedagogical approaches of physical education cross constant discussions about the best method for learning and developing students’ motor skills in relation to the teaching of collective modalities (34).

Although physical education training has undergone changes in recent years, the reference in terms of methodologies and pedagogical actions used by teachers currently includes, in many cases, the teaching of sport in the traditional approach. The methods used to implement the traditional approach, in which students are enabled to perform technical elements, are very far from what would be expected in terms of transfer to the game situation (35). For the technical skills to be transferred to a real game situation, the student must experience, from the very beginning of the learning process, a series of sequences that represent the game situations, i.e., it is necessary for the teacher to have the sensitivity to carry out tasks that are as close as possible to the “real” game. By deconstructing the game and creating situations of progression, the student will tend to bring a simplified tasks closer to the real situation, thus giving meaning to the learning. In collective sports, the game situation changes with each attack, so that technical skills are subject to rhythmic variations, intensity and amplitude. The aim of tactical learning is therefore for the student to learn to make decisions to solve problems at every turn, not to limit the success of the actions taken (36). Thus, and to observe and measure the students’ performance, it is necessary to instrumentalize the evaluation of team sports games in a school context. One of the most commonly used instruments for this purpose is the Game Performance Assessment Instrument (GPAI), which allows for the analysis of action outcome (i.e., movement product) and the motor execution process variables related to game actions (e.g., technique and tactics) (37).

As far as is known, no study yet has investigated the effects of a multivariate training program consisting of game-based activities and physical fitness exercises to improve physical and tactical performance in team sports (e.g., basketball), during physical education classes, and at different levels of education (basic and secondary education). Therefore, the main objective of this study was to verify the effects of applying a multivariate training program on physical fitness and tactical performance during the teaching of a basketball didactic unit in basic and secondary education. The study hypothesis is that the training program will have a positive effect on physical fitness and tactical performance in both teaching groups.

## 2. Methods

### 2.1. Participants

Seventy-five students from a Portuguese school participated in the study (15.02 ± 1.31 years) forty-two students from basic school education (Control group *n *= 21; Intervention Group *n *= 21) and thirty-three students from secondary school (Control group *n* = 17; Intervention Group *n* = 16). All subjects participated in the study voluntarily and their parents or guardians were informed in writing about the study and signed an informed consent form to confirm their child's participation. To participate in the study, the students needed to be in good health and regularly attend physical education classes, with no specific exclusion criteria in place. To calculate the sample, the G*Power 3.1 software was used for this quasi-experimental randomized controlled trial (38). It was established through an *a priori* analysis that 75 students would be required for the study (effect size dz: 0.3, error probability *α*: 0.05, power: 0.85). The students and the professor were informed about the genesis and requirements of the study, the risks involved, and the possibility of withdrawing from the research even after volunteering. All procedures followed the guidelines of the Declaration of Helsinki for research involving human subjects. The experimental approach was approved and followed by the local ethical committee (Project Number D2605).

### 2.2. Procedures

During the teaching of the basketball didactic unit (i.e., 6-weeks interventions), the students were randomly divided into two groups, a control group that will not carry out the training program and an experimental group that will carry out a training program consisting of strength, high-intensity explosive exercises and game-based activities. The two groups were assessed in two moments: before the application of the training program (pre-training test) and after the application of the training program (post-training test) in terms of changes in physical fitness and tactical performance. The experimental group began the multivariate training program class by class, while the control group began the warm-up at the same time for approximately 15 min.

Changes in the level of physical fitness were measured using the FITescola® test battery (i.e., sit-ups, push-ups, horizontal impulse, shuttle test, 40 m sprint and agility). Changes in tactical performance were measured using the GPAI instrument after collecting videos of match situations.

### 2.3. Training program

The training program was developed based on some adaptations of previous studies (22, 39). The training program was carried out over 6 weeks (Table 1) and consisted of four stations, detailed below, and 3 vs. 3 SSGs played in the midfield without the intervention of the coach and considering the knowledge acquired during the teaching of the didactic unit.

**Table 1:** Multivariate training program.

	S	R	Push-up	Change of direction	Horizontal impulse	Squat jump	Sprint	Russian twist	Jump	Plank shoulder taps	Max. sprint 4 × 10 M
Week 1	S1	2	4 × 10	4 × 8	4 × 4	4 × 10	4 × 1	4 × 20	4 × 5	4 × 20	4 × 1
Week 2	S2	2	4 × 10	4 × 8	4 × 4	4 × 10	4 × 1	4 × 20	4 × 5	4 × 20	4 × 1
	S3	2	4 × 10	4 × 8	4 × 4	4 × 10	4 × 1	4 × 20	4 × 5	4 × 20	4 × 1
Week 3	S4	2	4 × 10	4 × 8	4 × 4	4 × 10	4 × 1	4 × 20	4 × 5	4 × 20	4 × 1
	S5	3	6 × 10	6 × 8	6 × 4	6 × 10	6 × 1	6 × 20	6 × 5	6 × 20	6 × 1
Week 4	S6	3	6 × 10	6 × 8	6 × 4	6 × 10	6 × 1	6 × 20	6 × 5	6 × 20	6 × 1
	S7	3	6 × 10	6 × 8	6 × 4	6 × 10	6 × 1	6 × 20	6 × 5	6 × 20	6 × 1
Week 5	S8	3	6 × 10	6 × 8	6 × 4	6 × 10	6 × 1	6 × 20	6 × 5	6 × 20	6 × 1
	S9	4	8 × 10	8 × 8	8 × 4	8 × 10	8 × 1	8 × 20	8 × 5	8 × 20	8 × 1
Week 6	S10	4	8 × 10	8 × 8	8 × 4	8 × 10	8 × 1	8 × 20	8 × 5	8 × 20	8 × 1
	S11	4	8 × 10	8 × 8	8 × 4	8 × 10	8 × 1	8 × 20	8 × 5	8 × 20	8 × 1

R, repetitions; S, sessions.

#### 2.3.1. Station 1

Consists of two exercises: the 4 × 10 m maximum speed and 10 push-ups. For the maximum speed exercise, a 10-meter corridor was set up. The students were asked to complete the exercise at their maximum speed in the four lanes, and to move their feet beyond the signaling cones when changing direction (factors directly related to the FITescola® test). For the push-up exercise, the students were asked to straighten their torso and bend their arms at 90°.

#### 2.3.2. Station 2

Consists of three exercises: running with changes of direction, horizontal push (into the cones) and the squat jump. For the change of direction running exercise, eight flags were placed at equal intervals, and they had to be circled from the outside, at the maximum possible speed. For the horizontal push, two rows of arcs were placed (preferably different colors for each row) with one row of arcs at a greater distance than the other, depending on the level of difficulty to be applied. It is ideal to increase the distance as the session progresses, forcing the students to jump further. As for the squat jump, amplitude is required in both the squat and the vertical jump and should be as dynamic as possible.

#### 2.3.3. Station 3

Consists of two exercises: sprint and Russian twist. The sprint exercise is about 10 meters, so students should do it at maximum speed, with a start and finish flag. Students should start the exercise by trying to accelerate at the beginning of the race and maintain maximum speed until they pass the finish signal, not slowing down before then. The Russian twist exercise must be performed with the lower limbs raised (flexed), in a dynamic manner, with the abdomen turning sideways.

#### 2.3.4. Station 4

Consists of two exercises: Jump and Plank Shoulder Taps. The Jump exercise must be performed with feet together, without interruption, for the duration of the five hurdles. The height of the hurdles should also be adjusted (increased) throughout the sessions. The plank shoulder taps exercise must be performed in a plank position with the arms alternately touching the opposite shoulder with the hand, without breaking the starting position.

Students were asked to distribute themselves randomly (i.e., two to three students per station) to avoid queuing and to ensure that they were always exercising. The initial distribution was maintained throughout the application of the training program in Table 1.

### 2.4. Instruments

#### 2.4.1. Physical fitness

The FITescola® test battery was used to assess physical fitness (i.e., sit-ups, push-ups, horizontal impulse, shuttle test, 40 m sprint, agility 4 × 10 m). The tests used are described in detail below.

#### 2.4.2. Sit-ups

The sit-up test consists of performing as many sit-ups as possible at a given cadence (40). The purpose of this test is to assess the resistance of the abdominal muscles. This action was repeated at a predetermined cadence of 20 sit-ups per minute. The number of times the participants performed this action correctly was recorded.

#### 2.4.3. Push-ups

The push-up test consists of performing the maximum number of push-ups (arm flexion and forearm extension) at a predetermined cadence (40). This test aims to assess the endurance strength of the upper limbs. This action was repeated at a predetermined cadence of 20 push-ups per minute. The number of correctly performed actions was subsequently recorded.

#### 2.4.4. Horizontal impulse

The horizontal impulse test consists of reaching the maximum distance in a long jump with feet together (40). The purpose of this test is to assess the explosive strength of the lower limbs. A horizontal line is drawn at the starting point, with reference lines every 10 cm. Starting from a standing position, the subject must bend the knees, pull the arms behind him and jump in length as far as possible. Two jumps must be made. The best of the two measurements is recorded in cm.

#### 2.4.5. Shuttle test

The shuttle test consists of completing the maximum number of routes performed over a distance of 20 m, at a predetermined cadence, using an audio device (41). The student must remain in the test for as long as possible and must stop if they fail to cross the line before the buzzer on two occasions, not necessarily in a row. The first miss is counted towards the score.

#### 2.4.6. 40 m sprint

The test consists of running a 40 m race in the shortest possible time according to a previously described protocol (42). The purpose of this test is to measure the student's acceleration capacity and speed. There must be two attempts per student. The recorded value is the best result of the two trials, in hundredths of second.

#### 2.4.7. Agility 4 × 10m

The agility test (4 × 10 m) consists of completing a predetermined route, combining maximum speed of execution with coordination translated into the movement of grasping, carrying and placing a sponge in a predetermined place (43). The purpose of the test is to assess the student's agility, characterizing the ability to accelerate, the coordination of the movements required and the speed at which they are performed. Two tests must be carried out and the registered value is the best result of the two evaluations, expressed in hundredths.

#### 2.4.8. Game performance assessment instrument (GPAI)

*GPAI:* The GPAI was used to assess students’ tactical performance in accordance with previously reported recommendations (44). Each player's game performance in a 20-minute 3 vs*.* 3 match was coded by two experts using footage that was taken with a standard film camera. The experts were selected based on the following criteria: (i) having at least 10 years of professional experience in the modality; (ii) current experience as a high-performance basketball coach; (iii) at least a Master's degree with scientific research in the modality. The GPAI record sheet was used by the experts during the assessment. In order to validate the observations, coders had to achieve a coefficient of inter-observer agreement greater than 80% using the Kendall's W coefficient of agreement (44). Coders recorded and calculated measures of appropriate and inappropriate actions in four components of the game, taking into account the Decision Making Index (DMI), the Skill Execution Index (SEI), the Support Actions Index (SI), and the Adaptability Index (AI), based on an adaptation of a previously defined and validated protocol (44) (Table 2).

**Table 2:** Definition of appropriate and inappropriate actions for applying the GPAI in the following indexes: decision making index (DMI), skill execution index (SEI), support actions (SI) and adaptability index (AI).

Components of game	Assumption	AA	IA
Decision-making	At reception, he fits in with the basket in a basic offensive posture.	Fulfils the assumption	Does not meet the assumption
	Shoots when he is within range of the basket and the defender is not pressing him.		
	Passes when he has an unmarked teammate in a more offensive position		
	If he has none of the above options, he dribbles away from the defender.		
Skill execution	Throwing: Throwing arm extension, wrist flexion.		
	Passing: Passing to a teammate; the ball reaches the teammate in good condition.		
	Dribbling: Does not look at the ball; does not “carry” the ball.		
Support actions	Attempts to create passing lines.		
	Does not come within 3 m of the person with the ball.		
Saving/marking	When he loses the ball, he takes a basic offensive stance and looks for his immediate opponent.		
	Places himself between his opponent and the basket.		

AA, appropriate action; IA, inappropriate action.

These four game components were chosen as the most important for assessing general and non-specific game skills in young people.

### 2.5. Statistical analysis

Descriptive statistics were presented as the mean ± one standard deviation (SD) with a 95% confidence interval (CI). The Shapiro–Wilk and Levene tests were used to determine the normality and homogeneity of the data distribution. The independent samples *t*-test (samples from two groups) and paired sample test (for the same group) were used to compare appropriate and inappropriate actions within each group and between groups before and after the intervention. Effect sizes (ES) were calculated based on Cohen's d and classified as: 0.2–0.6 (trivial); 0.6–1.2 (small); 1.2 (large); and greater than 2.0 (very large). Mean differences (Δ) were presented in absolute values (45, 46). Statistical significance was set at *p* < 0.05. Statistical analyses were performed using SPSS for Windows Version 26.0 (SPSS Inc., Chicago, IL, USA).

## 3. Results

### 3.1. Pre-post assessments for GPAI indexes and physical fitness in all groups (SS, secondary school and BS, Basic school)

Table 3 shows the comparison between the Pre-Post assessment for GPAI indices in all groups (SS and BS). All indexes present significant differences between methods with small effects (*t* = −6.54 to −4.82, *Δ* = −27.57 to −0.16, *p* < 0.05–*p* < 0.001, *d* = 0.78 to 1.05).

**Table 3:** Pre-Post Assessment for GPAI indexes in all groups (SS and BS).

Variable	Pre_Overall_	Post_Overall_	*t*	Δ	*p*	*d*	*QS*
GI	45.52 ± 23.52	73.09 ± 28.59	−6.54	−27.57	<0.001	1.05	Small
DMI	0.72 ± 0.24	0.90 ± 0.14	−5.67	−0.18	<0.001	0.91	Small
SEI	0.49 ± 0.31	0.75 ± 0.24	−5.79	−0.26	<0.001	0.93	Small
SI	0.69 ± 0.23	0.84 ± 0.17	−4.82	−0.16	<0.001	0.78	Small
AI	0.57 ± 0.24	0.73 ± 0.14	−5.35	−0.17	<0.001	0.86	Small
GP	0.63 ± 0.22	0.83 ± 0.15	−6.42	−0.20	<0.001	1.04	Small

Δ, mean differences; AI, adaptability index; *d*, cohen; DMI, Decision Making Index; GP, game performance; *p*, *p* value; *QS*, qualitative effect size; SEI, skill execution index; SI, support index; GI, game involvement; *t*, *t*-test.

Table 4 shows the comparison between the Pre-Post assessment for physical fitness in all groups (SS and BS). Significant differences with trivial effects were found between the pre- and post-assessment for the speed test (*t* = 2.27, *Δ* = 0.30, *p* = 0.025, *d* = 0.37) and the agility test (*t* = 3.09, *Δ* = 0.62, *p* = 0.002, *d* = 0.51).

**Table 4:** Pre-Post assessment of physical fitness in all groups (SS and BS).

Variable	Pre_Overall_	Post_Overall_	*t*	Δ	*p*	*d*	*QS*
Horizontal jump	151.76 ± 36.40	161.14 ± 34.03	−1.62	−9.38	0.108	0.27	Trivial
Velocity (40 m)	7.16 ± 0.77	6.87 ± 0.82	2.27	0.30	0.025	0.37	Trivial
Agility (4 × 10)	13.10 ± 1.24	12.47 ± 1.21	3.09	0.62	0.002	0.51	Trivial
Abdominal strength	43.73 ± 21.84	48.38 ± 24.47	−1.28	−4.65	0.204	0.21	Trivial
Shuttle run (20 m)	36.37 ± 20.15	38.77 ± 20.50	−0.72	−2.41	0.473	0.12	Trivial
Push ups	12.62 ± 8.03	14.37 ± 8.26	−1.30	−1.74	0.195	0.21	Trivial

Δ, mean differences; *d*, cohen; *p*, *p* value; *QS*, qualitative effect size; *t*, *t*-test.

### 3.2. Inter-group comparison for pre- and post-assessment moments in basic school

Table 5 shows inter-group comparison for the pre and post assessment moment (Pre CG vs. Pre IG and Post CG vs. Post IG, respectively) for the GPAI indexes in basic school students. The pre-tests showed no significant differences for the overall GPAI indexes. Otherwise, significant differences with small effect sizes were found in the post-tests for the DMI (*t* = −3.13, *Δ**** =* **−0.07, *p* = 0.003, *d* = 0.97) and SI (*t* = −2.38, *Δ**** =* **−0.12, *p* = 0.022, *d* = 0.74).

**Table 5:** Inter-group comparison for the Pre and post assessment moment (Pre CG vs. Pre IG and Post CG vs. Post IG, respectively) for the GPAI indexes in basic school students.

Variable	Pre _CG_	Pre _IG_	*t*	Δ	*P*	*d*	*QS*	Post _CG_	Post _IG_	*t*	Δ	*p*	*d*	*QS*
GI	44.24 ± 25.98	41.95 ± 20.16	0.319	2.29	0.752	0.10	Trivial	58.95 ± 31.26	78.86 ± 32.81	−2.01	−19.91	0.051	0.62	Small
DMI	0.74 ± 0.21	0.73 ± 0.29	0.148	0.01	0.883	0.05	Trivial	0.93 ± 0.09	0.99 ± 0.02	−3.13	−0.07	0.003	0.97	Small
SEI	0.43 ± 0.31	0.52 ± 0.38	−0.850	−0.09	0.400	0.26	Trivial	0.70 ± 0.29	0.77 ± 0.27	−0.82	−0.07	0.416	0.25	Trivial
SI	0.67 ± 0.22	0.79 ± 0.17	−1.994	−0.12	0.053	0.62	Small	0.79 ± 0.22	0.91 ± 0.09	−2.38	−0.12	0.022	0.74	Small
AI	0.55 ± 0.23	0.54 ± 0.24	0.086	0.01	0.932	0.03	Trivial	0.69 ± 0.15	0.71 ± 0.14	−0.58	−0.03	0.563	0.18	Trivial
GP	0.61 ± 0.23	0.68 ± 0.25	−0.894	−0.07	0.377	0.28	Trivial	0.80 ± 0.18	0.89 ± 0.11	−1.88	−0.09	0.068	0.58	Trivial

Δ, mean differences; AI, adaptability index; *d*, cohen; DMI, Decision Making Index; GP, game performance; *p*, *p* value; *QS*, qualitative effect size; SEI, skill execution index; SI, support index; GI, game involvement; *t*, *t*-test.

Regarding the effects of the training program on physical fitness, differences were found between the CG and the IG in the pre-test comparisons (Table 6). Particularly, significant differences with small effects were found for horizontal jump (*t* = −3.26, *Δ* = −26.76, *p* = 0.002, d = 1.01), agility (*t *= 3.35, *Δ* = 1.29, *p* = 0.002, *d* = 1.04) and shuttle run (*t* = 2.27, *Δ* = 12.86, *p* = 0.029, *d *= 0.70). Post-tests showed significant differences for horizontal jump (*t* = −3.39, *Δ* = −26.43, *p* = 0.002, *d* = 1.05) and agility (*t *= 2.31, *Δ* = 0.86, *p* = 0.026, *d* = 0.71).

**Table 6:** Inter-group comparison for the pre and post assessment moment (Pre CG vs. Pre IG and Post CG vs. Post IG, respectively) for the physical fitness in basic school students.

Variable	Pre _CG_	Pre _IG_	*t*	Δ	*P*	*d*	*QS*	Post _CG_	Post _IG_	*t*	Δ	*p*	*d*	*QS*
Horizontal jump	137.10 ± 26.21	163.86 ± 26.92	−3.26	−26.76	0.002	1.01	Small	134.81 ± 22.91	161.14 ± 27.40	−3.39	−26.43	0.002	1.05	Small
Velocity (40 m)	7.43 ± 0.68	7.05 ± 0.67	1.84	0.38	0.074	0.57	Trivial	7.14 ± 0.85	6.71 ± 0.85	1.64	0.43	0.110	0.51	Trivial
Agility (4 × 10)	13.86 ± 1.24	12.57 ± 1.25	3.35	1.29	0.002	1.04	Small	12.76 ± 1.41	11.91 ± 0.94	2.31	0.86	0.026	0.71	Small
Abdominal strength	47.81 ± 23.66	44.57 ± 26.01	0.42	3.24	0.675	0.13	Trivial	58.29 ± 21.31	53.76 ± 25.71	0.62	4.52	0.538	0.19	Trivial
Shuttle run (20 m)	40.24 ± 20.86	27.38 ± 15.46	2.27	12.86	0.029	0.70	Trivial	32.95 ± 15.14	41.67 ± 24.14	−1.40	−8.71	0.169	0.43	Trivial
Push ups	10.52 ± 6.71	14.05 ± 8.16	−1.53	−3.52	0.134	0.47	Trivial	13.33 ± 17.49	17.14 ± 7.45	−1.65	−3.81	0.106	0.51	Trivial

Δ, mean differences; *d*, cohen; *p*, *p* value; *QS*, qualitative effect size; *t*, *t*-test.

### 3.3. Inter-group comparison for the pre- and post-assessment moments in secondary school

Table 7 shows the inter-group comparison for the pre- (Pre _CG_ vs. Pre _IG_) and post-assessment moments (Post _CG_ vs. Post _IG_) for the GPAI indexes in secondary school students. Significant differences with small to large effects were found in the pre-test assessment for DMI (*t* = −7.33, *Δ* = −0.36, *p* < 0.001, *d* = 2.49), SI (*t* = −3.00, *Δ* = −0.24, *p* = 0.005, *d *= 1.02), AI (*t *= −4.08, *Δ* = −0.28, *p* < 0.001, *d* = 1.38) and GP (*t* = −3.22, *Δ* = −0.20, *p* = 0.003, *d* = 1.09). In the post-tests, significant differences with small to very large effects were found for the overall indexes (*t* = −8.63 to 2.83, *Δ* = −0.26–18.52, *p *<* *0.05 to *p *<* *0.001, *d* = 0.96 to 2.93).

**Table 7:** Inter-group comparison for the pre- and post-assessment moment (Pre CG vs. Pre IG and Post CG vs. Post IG, respectively) for the GPAI indexes in secondary school students.

Variable	Pre _CG_	Pre _IG_	*t*	Δ	*p*	*d*	*QS*	Post _CG_	Post _IG_	*t*	Δ	*P*	*d*	*QS*
GI	49.79 ± 26.84	46.81 ± 21.23	0.36	2.98	0.722	0.12	Trivial	86.58 ± 19.85	68.06 ± 18.62	2.83	18.52	0.008	0.96	Small
DMI	0.53 ± 0.18	0.89 ± 0.09	−7.33	−0.36	<0.001	2.49	Very large	0.71 ± 0.11	0.96 ± 0.04	−8.63	−0.26	<.0001	2.93	Very large
SEI	0.51 ± 0.18	0.52 ± 0.34	−0.11	−0.01	0.917	0.04	Trivial	0.66 ± 0.15	0.90 ± 0.12	−5.07	−0.23	<0.001	1.72	Very large
SI	0.53 ± 0.23	0.76 ± 0.24	−3.00	−0.24	0.005	1.02	Small	0.74 ± 0.15	0.95 ± 0.06	−5.12	−0.21	<0.001	1.74	Very large
AI	0.47 ± 0.22	0.75 ± 0.18	−4.08	−0.28	<0.001	1.38	Large	0.70 ± 0.09	0.87 ± 0.08	−5.53	−0.16	<0.001	1.88	Very large
GP	0.52 ± 0.18	0.73 ± 0.20	−3.22	−0.20	0.003	1.09	Small	0.70 ± 0.11	0.94 ± 0.05	−7.57	−0.23	<0.001	2.57	Very large

Δ, mean differences; d, cohen; DMI, Decision Making Index; GP, game performance; *p*, *p* value; *QS*, qualitative effect size; SEI, skill execution index; SI, support index; GI, game involvement; *t*, *t*-test.

Regarding the effects of the training program on physical fitness, no differences were found between the CG and the IG in the inter-group comparison between pre- and post-test assessments (Table 8).

**Table 8:** Inter-group comparison for the pre and post assessment moment (Pre CG vs. Pre IG and Post CG s. Post IG respectively) for physical fitness in secondary school students.

Variable	Pre _CG_	Pre _IG_	*t*	Δ	*p*	*d*	*QS*	Post _CG_	Post _IG_	*t*	*Δ*	*p*	*d*	*QS*
Horizontal jump	157.24 ± 50.51	149.13 ± 37.31	0.51	8.10	0.614	0.18	Trivial	174.24 ± 34.95	183.00 ± 32.58	−0.73	−8.77	0.471	0.26	Trivial
Velocity (40 m)	7.06 ± 1.03	7.07 ± 0.70	−0.03	−0.01	0.980	0.01	Trivial	6.65 ± 0.86	6.93 ± 0.59	−1.08	−0.29	0.289	−0.38	Trivial
Agility (4 × 10)	12.71 ± 0.92	13.20 ± 1.08	−1.40	−0.49	0.173	0.50	Trivial	12.41 ± 0.94	12.93 ± 1.28	−1.33	−0.52	0.195	0.47	Trivial
Abdominal strength	44.94 ± 16.53	35.47 ± 17.55	1.57	9.48	0.126	0.56	Trivial	38.24 ± 15.88	38.47 ± 18.18	−0.04	−0.23	0.970	0.01	Trivial
Shuttle run (20 m)	43.53 ± 23.62	35.40 ± 17.61	1.09	8.13	0.284	0.39	Trivial	42.71 ± 20.42	38.40 ± 21.71	0.58	4.31	0.568	0.21	Trivial
Push ups	14.59 ± 8.11	11.33 ± 9.26	1.06	3.26	0.297	0.38	Trivial	12.00 ± 9.05	14.60 ± 9.09	−0.81	−2.60	0.425	0.29	Trivial

Δ, mean differences; *d*, cohen; *p*, *p* value; *QS*, qualitative effect size; *t*, *t*-test.

### 3.4. Intra-group comparison (CG, control group vs. IG, intervention group) in basic school

Considering the effects of the training program on tactical performance, an intra-group comparison was made between the pre- and post-assessment for the GPAI indexes (Table 9). Significant differences with trivial to large effect sizes were found between pre- and post-tests for the CG (*t* = −3.71 to −1.66; *Δ* = −14.71 to −0.14, *p* < 0.05 *p* < 0.001, *d* = 0.51–1.15) and the IG (*t* = −4.39 to −2.48; *Δ* = −36.91 to −0.12, *p* < 0.05 *p* < 0.001, *d* = 0.77–1.36), except for GI and SI in CG. Considering the effects of the training program on physical fitness, an intra-group comparison was made between the pre- and post-assessment tests (Table 10). In the CG, significant differences with trivial effect sizes were found between the pre- and post-tests for agility (*t* = 2.68, *Δ* = 0.29, *p* = 0.011, *d *= 0.83). In the IG, significant differences with trivial effects were reported for shuttle run (*t* = −2.28, *Δ* = −14.29, *p* = 0.028, *d *= 0.71).

**Table 9:** Intra-group comparison (CG and IG) in the pre and post assessments for the GPAI indexes in basic school.

	Control group (CG)							Intervention group (IG)						
Variable	Pre	Post	*t*	Δ	*p*	*d*	*QS*	Pre	Post	*t*	Δ	*p*	*d*	*QS*
GI	44.24 ± 25.98	58.95 ± 31.26	−1.66	−14.71	0.105	0.51	Trivial	41.95 ± 20.16	78.86 ± 32.81	−4.39	−36.91	<0.001	1.36	Large
DMI	0.74 ± 0.21	0.93 ± 0.09	−3.71	−0.19	<0.001	1.15	Small	0.73 ± 0.29	0.99 ± 0.02	−4.11	−0.27	<0.001	1.27	Large
SEI	0.43 ± 0.31	0.70 ± 0.29	−2.94	−0.27	0.005	0.91	Small	0.52 ± 0.38	0.77 ± 0.27	−2.48	−0.25	0.017	0.77	Small
SI	0.67 ± 0.22	0.79 ± 0.22	−1.75	−0.12	0.088	0.54	Small	0.79 ± 0.17	0.91 ± 0.09	−2.88	−0.12	0.006	0.89	Small
AI	0.55 ± 0.23	0.69 ± 0.15	−2.34	−0.14	0.025	0.72	Trivial	0.54 ± 0.24	0.71 ± 0.14	−2.84	−0.17	0.007	0.88	Small
GP	0.61 ± 0.23	0.80 ± 0.18	−3.01	−0.19	0.005	0.93	Small	0.68 ± 0.25	0.89 ± 0.11	−3.61	−0.21	<0.001	1.11	Small

DMI, Decision Making Index; SEI, skill execution index; SI, support index; GI, game involvement; GP, game performance; *d*, cohen; Δ, mean differences; *p*, *p* value.

**Table 10:** Intra-group comparison (CG and IG) in the pre and post assessments for physical fitness in basic school.

	Control group (CG)							Intervention group (IG)						
Variable	Pre	Post	*t*	Δ	*p*	*d*	*QS*	Pre	Post	*t*	Δ	*p*	*d*	*QS*
Horizontal jump	137.10 ± 26.21	134.81 ± 22.91	0.30	−0.71	0.765	0.09	Trivial	163.86 ± 26.92	161.24 ± 27.40	0.31	2.62	0.756	0.10	Trivial
Velocity (40 m)	7.43 ± 0.68	7.14 ± 0.85	1.20	2.29	0.236	0.37	Trivial	7.05 ± 0.67	6.71 ± 0.85	1.48	0.33	0.164	0.44	Trivial
Agility (4 × 10)	13.86 ± 1.24	12.76 ± 1.41	2.68	0.29	0.011	0.83	Trivial	12.57 ± 1.25	11.91 ± 0.94	1.95	0.67	0.058	0.60	Trivial
Abdominal strength	47.81 ± 23.66	58.29 ± 21.31	−1.51	1.10	0.139	0.47	Trivial	44.57 ± 26.01	53.76 ± 25.71	−1.15	−9.19	0.256	0.36	Trivial
Shuttle run (20 m)	40.24 ± 20.86	32.95 ± 15.14	1.30	−10.48	0.203	0.40	Trivial	27.38 ± 15.46	41.68 ± 24.14	−2.28	−14.29	0.028	0.71	Trivial
Push ups	10.52 ± 6.71	13.33 ± 7.49	−1.28	7.29	0.208	0.40	Trivial	14.05 ± 8.16	17.14 ± 7.45	−1.28	2.62	0.207	0.40	Trivial

Δ, mean differences; *d*, cohen; *p*, *p* value; *QS*, qualitative effect size; *t*, *t*-test.

### 3.5. Intra-group comparison (CG, control group vs. IG, intervention group) in secondary school

Considering the effects of the training program on tactical performance, an intra-group comparison was made between the pre- and post-assessment for the GPAI indexes (Table 11). Significant differences with small to large effect sizes were found between the pre- and post-tests in both groups, specifically: CG (*t* = −4.80 to −2.77; *Δ* = −36.79 to −0.15, *p* < 0.05 t o *p *< 0.001, *d* = 0.90–1.56) and IG (*t* = −4.15 to −2.39; *Δ* = −21.25 to −0.07, *p* < 0.05 to *p *< 0.001, *d* = 0.99–1.47).

**Table 11:** Intra-group comparison (CG and IG) in the pre and post assessments for the GPAI indexes in secondary school.

	Control group (CG)							Intervention group (IG)						
Variable	Pre	Post	*t*	Δ	*p*	*d*	*QS*	Pre	Post	*t*	Δ	*p*	*d*	*QS*
GI	49.79 ± 26.84	86.58 ± 19.85	−4.80	−36.79	<0.001	1.56	Large	46.81 ± 21.23	68.06 ± 18.62	−3.01	−21.25	0.005	1.06	Small
DMI	0.53 ± 0.18	0.71 ± 0.12	−3.57	−0.18	0.001	1.16	Small	0.90 ± 0.09	0.96 ± 0.04	−2.79	−0.07	0.009	0.99	Small
SEI	0.51 ± 0.18	0.66 ± 0.15	−2.77	−0.15	0.009	0.90	Small	0.52 ± 0.34	0.90 ± 0.12	−4.12	−0.37	<0.001	1.46	Large
SI	0.52 ± 0.18	0.74 ± 0.15	−3.45	−0.22	0.001	1.12	Small	0.76 ± 0.24	0.95 ± 0.06	−3.02	−0.19	0.005	1.07	Small
AI	0.47 ± 0.22	0.70 ± 0.09	−4.33	−0.23	<0.001	1.41	Large	0.75 ± 0.18	0.87 ± 0.08	−2.39	−0.12	0.023	0.85	Small
GP	0.52 ± 0.18	0.70 ± 0.11	−3.72	−0.18	<0.001	1.21	Large	0.73 ± 0.20	0.94 ± 0.05	−4.15	−0.21	<0.001	1.47	Large

DMI, Decision Making Index; SEI, skill execution index; SI, support index; GI, game involvement; GP, game performance; *d*, cohen; Δ, mean differences; *p*, *p* value.

Considering the effects of the training program on physical fitness, an intra-group comparison was made between the pre- and post-assessment tests (Table 12). In the GC, no statistical significance was found between the pre and post physical fitness. In the IG, significant differences were reported for the horizontal jump (*t* = −2.65, *Δ* = −33.87, *p* = 0.013, *d *= 0.97).

**Table 12:** Intra-group comparison (CG and IG) in the pre and post assessments for physical fitness in secondary school.

	Control group (CG)							Intervention group (IG)						
Variable	Pre	Post	*t*	Δ	*p*	*d*	*QS*	Pre	Post	*t*	Δ	*p*	*d*	*QS*
Horizontal jump	157.24 ± 50.51	174.24 ± 34.95	−1.14	−17.00	0.262	0.39	Trivial	149.13 ± 37.31	183.00 ± 32.56	−2.65	−33.87	0.013	0.97	Small
Speed (40 m)	7.06 ± 1.03	6.65 ± 0.86	1.27	0.41	0.215	0.43	Trivial	7.07 ± 0.70	6.93 ± 0.59	0.56	0.13	0.579	0.21	Trivial
Agility (4 × 10)	12.71 ± 0.92	12.41 ± 0.94	0.92	0.29	0.363	0.32	Small	13.20 ± 1.08	12.93 ± 1.28	0.62	0.27	0.543	0.23	Trivial
Abdominal strength	44.94 ± 16.53	38.24 ± 15.88	1.21	6.71	0.237	0.41	Small	35.47 ± 17.55	38.47 ± 18.18	−0.46	−3.00	0.649	0.17	Trivial
Shuttle run (20 m)	43.53 ± 23.62	42.71 ± 20.42	0.11	0.82	0.914	0.04	Trivial	35.40 ± 17.61	38.40 ± 21.71	−0.42	−3.00	0.681	0.15	Trivial
Push ups	14.59 ± 8.11	12.00 ± 9.05	0.88	2.59	0.386	0.30	Trivial	11.33 ± 9.26	14.60 ± 9.09	−0.98	3.10	0.338	0.40	Trivial

Δ, mean differences; *d*, cohen; *p*, *p* value; *QS*, qualitative effect size; *t*, *t-*test.

## 4. Discussion

The aim of the study was to investigate the effects of a 6-week multivariate training program on physical fitness and tactical performance during a basketball didactic unit in basic and secondary schools. The results of the study showed that there was a significant difference between the pre- and post-test for all GPAI indices in both groups, indicating an improvement in tactical performance. The implementation of a multivariate program in basic and secondary schools also has a positive effect, especially on variables related to game performance. There were significant differences between the pre- and post-test scores for decision-making (DMI) and support actions (SI) for Basic school students. The secondary school students showed a significant difference between all GPAI scores on the post-test. Regarding physical fitness, the multivariate training program had some positive effects on physical fitness, especially on the speed and agility components. However, there were no other significant differences in physical fitness variables in basic and secondary school students. In a school environment, a well-structured multivariate training program can effectively improve students’ tactical skills while increasing their physical fitness levels.

Overall, differences were found in all GPAI variables for basic and secondary school students. These results indicate that the teaching of the didactic unit had a positive effect on Game Involvement (GI) Decision-Making (DMI), Skill Execution (SEI), Support Actions (SI), Adaptability (AI) and overall Game Performance (GP). The teaching-learning process was also positive in the areas of motor skill execution and game understanding. Silva et al. (47) concluded that the use of a multivariate training program can bring benefits in several areas of student development, such as physical fitness, creativity and motor skills. A tactical approach can be useful to improve students’ Decision-Making, Skill Execution, Game Involvement (48–51), Adaptability (52), Support Actions and overall Game Performance (50, 53). According to these studies, teaching a didactic unit with a multivariate approach focusing on tactical components can be very helpful. Some significant differences were observed in the speed (40 m) and agility (4 × 10) variables, suggesting that the training program significantly improved the participants' speed and agility, as evidenced by the significant differences observed in the 40-meter speed and 4 × 10 agility tests. However, the training program had no statistically significant effect on variables such as horizontal jump, abdominal strength, shuttle run (20 m), and push-ups. Different components of physical fitness may respond differently to training stimuli, and some participants may already have higher baseline values. In addition, the maturity, duration and intensity of the participants’ exercise programs may have influenced the observed differences in physical fitness variables. Some authors acknowledge that the effectiveness of a particular exercise program may vary greatly from person to person (54). Many individual factors such as training program characteristics, environmental conditions, regular physical activity, fitness level, physiological and genetic differences, and social and psychological factors contribute to the differences (54). In particular, the study found differences in all GPAI variables in basic and secondary school students. However, for physical fitness, the training program showed significant differences only for basic school students. In the context of basic education, specific differences were observed in the Decision Making Index (DMI) and the Support Index (SI). The observed increase in DMI and SI scores suggests that the instructional intervention had an impact on students’ decision-making and support skills, enabling them to make more effective decisions during the game, such as creating more passing lines to help teammates.

In terms of physical fitness, a previous study also showed that footballers with a higher physical fitness ratio demonstrated a greater number of skills, such as dribbling and shooting, during an 8-a-side football match game. The authors also observed a positive relationship between physical performance and player engagement throughout the game (55). In this study, the intervention had a positive effect on the students’ performance and engagement during the game. Multivariate training programs offer the opportunity to improve physical fitness, motor skills, and creativity. According to the results obtained, the multivariate training program tested seems to be an excellent tool and a pedagogical alternative for the integrated development of tactical indicators such as comprehension, interpretation and technical/tactical performance. A study conducted by Silva et al. (47) suggests that this type of training program is a valuable tool that can be used effectively for the simultaneous development of basic skills. They contribute to the development of different aspects, including technical, tactical, physical, and physiological factors (56). These training exercises provide players with a multidimensional experience that includes technical and tactical skills, as well as physiological and physical demands. Additionally, engaging in SSG allows players to focus on specific tactical elements while improving their overall understanding of the game and physical performance (57).

In this study, differences were observed between the Control and Intervention Groups in secondary school students on variables such as Game Involvement (GI), Skill Execution Index (SEI), Support Index (SI), Adaptability Index (AI), and Game Performance (GP). These differences were characterized by effect sizes ranging from trivial and small for SEI, SI and GP to very large for all variables, except for GI. In the post-test assessment of Game Involvement (GI), Decision-Making (DMI), Support Actions (SI), Adaptability (AI), and overall Game Performance (GP), both basic and secondary school students showed significant differences between the Control and Intervention Groups. However, the effect size was larger for secondary school students, suggesting that the training program had a greater impact on improving their game involvement, decision-making and overall game performance compared to basic school students. In terms of skill execution (SEI), secondary school students showed more significant differences between the Control and Intervention Groups in the post-test assessment compared to basic school students.

Considering the physical fitness variables, it is clear that the observed results may be influenced by individual variability resulting from different maturation processes and the interrelated development of motor skills across different tasks and exercises in different lessons, in an integrated manner and in relation to the different learning tasks. Individual differences in skill level and learning capacity between participants may have influenced the extent of progress in certain tactical aspects, leading to this variability. In addition, the duration and intensity of the training program may have had different effects on different GPAI variables (58, 59). Furthermore, the positive impact of other learning tasks in the classroom, not just the training program, should also be considered. However, the multivariate training program tested seems to be an excellent tool and a pedagogical alternative for the integrated development of tactical indicators such as understanding, interpretation and technical/tactical execution of the game. This type of program, used at the beginning of the physical education lesson as an alternative to the traditional warm-up, seems to allow for a holistic and well-rounded educational experience for the students, maximizing the useful class time and contributing to their overall development according to the objectives of the didactic unit (60). There is also evidence suggesting that the use of this type of training during adolescence is highly beneficial, as it allows the body to be trained during a period of accelerated musculoskeletal growth, at a time when balance and coordination are impaired as a result of this growth (19). Additionally, physical tests in basketball physical education provide essential quantitative data on an athlete's physical fitness, encompassing aspects like speed, agility, endurance, vertical leap, and strength.

Also confirming the potential of the multivariate program in terms of game performance, the students showed a significant improvement in their ability to make effective decisions during the game. The GPAI were based on critical game components such as decision-making, spatial awareness, teamwork, goals and strategic execution (48–51). Progress in decision-making skills reinforces the impact of the training program, which is also reflected in the positive results of the SEI. The increased SEI scores within the IG appear to be improving their skills. Moreover, the increase in SI scores, even considering the reduced 3 vs. 3 format, suggests that the IG improved in important aspects such as collective organization and the ability to create passing lines and help their teammates. The progress observed in the GP index was also supported by an increase in the overall skills of the GI, which enabled them to perform better during matches. Combining GPAI dimensions with physical tests offers a comprehensive assessment approach by qualitative insights into game performance with quantitative data on physical capabilities (54). This combined approach enables a well-rounded evaluation, aiding in tailored training programs, targeted skill development, and enhanced overall performance in basketball (58, 59).

This is a positive indicator of the positive association for match performance when considering the implementation of the training program. There may not be significant differences in all the physical fitness variables, but the functional significance for match improvement may come from the implementation of this multivariate approach. This study is not free of limitations, as it was not possible to control the potential impact that students’ extracurricular activities may have on outcomes, and future studies should take this variable into account (58, 59). The study results were not adjusted for baseline values, such as physical fitness levels, and no comparisons between genders or participant maturation status were done. Future studies on the Game Performance Assessment Instrument (GPAI) should focus on refining its metrics to accurately capture game scenarios and player roles, enhancing its validity and reliability (50, 53). Validating GPAI through correlations with actual game outcomes will establish its predictive value and practical relevance. Integration of cutting-edge technologies like AI and advanced analytics could automate data collection and analysis, making the GPAI more efficient and providing real-time insights (48–51). Additionally, exploring how GPAI-guided training programs impact individual and team performance will contribute to its broader adoption in sports coaching and talent development (52).

## 5. Conclusion

In conclusion, the study demonstrated that the implementation of a 6-week multivariate training program had positive effects on both physical fitness and tactical basketball performance in basic and secondary school students. The program led to significant improvements in tactical performance, particularly in Decision-Making, Skill Execution, Adaptability, Support Actions, and overall Game Performance. It also had a positive effect on physical fitness, particularly the speed and agility components. However, the program did not show statistically significant effects on all physical fitness variables such as horizontal jump, abdominal strength, shuttle run, and push-ups. Individual factors such as baseline fitness levels and the duration and intensity of the program may have influenced these results. Overall, the results suggest that a well-structured multivariate training program can effectively improve the tactical skills and physical fitness of students in a school setting.

## Data Availability

The raw data supporting the conclusions of this article will be made available by the authors, without undue reservation.
